# Toward Complete All-Optical Intensity Modulation of High-Harmonic Generation from Solids

**DOI:** 10.1021/acsphotonics.4c00156

**Published:** 2024-04-24

**Authors:** Pieter J. van Essen, Zhonghui Nie, Brian de Keijzer, Peter M. Kraus

**Affiliations:** † Advanced Research Center for Nanolithography, Science Park 106, 1098 XG Amsterdam, The Netherlands; ‡ Department of Physics and Astronomy, and LaserLaB, Vrije Universiteit, De Boelelaan 1105, 1081 HV Amsterdam, The Netherlands

**Keywords:** high-harmonic generation, attosecond science, condensed matter, ultrafast dynamics, insulator-to-metal transition, emission control

## Abstract

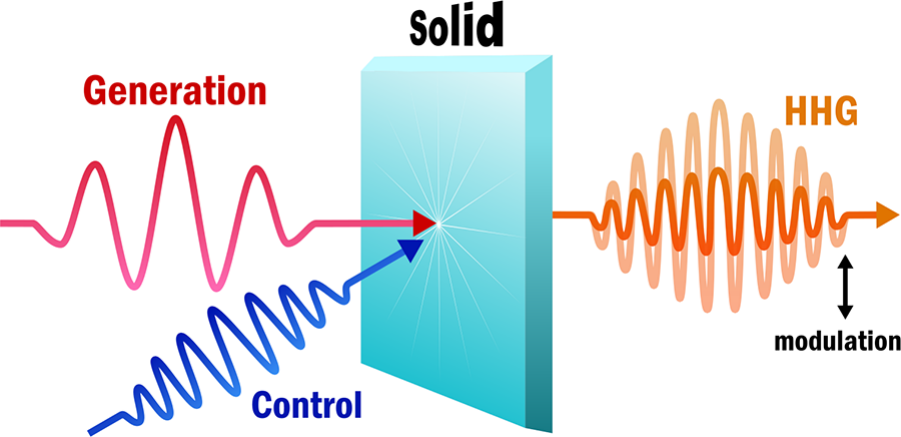

Optical modulation of high-harmonics generation in solids enables the detection of material properties, such as the band structure, and promising new applications, such as super-resolution imaging in semiconductors. Various recent studies have shown optical modulation of high-harmonics generation in solids, in particular, suppression of high-harmonics generation has been observed by synchronized or delayed multipulse sequences. Here we provide an overview of the underlying mechanisms attributed to this suppression and provide a perspective on the challenges and opportunities regarding these mechanisms. All-optical control of high-harmonic generation allows for femtosecond, and in the future possibly subfemtosecond, switching, which has numerous possible applications: These range from super-resolution microscopy to nanoscale controlled chemistry and highly tunable nonlinear light sources.

## 1 Introduction

High-harmonic generation (HHG) allows for the coherent generation of ultrashort pulses, which has enabled the field of attosecond science for the study of ultrafast phenomena,^1−7^ nanoscale microscopy via coherent diffraction imaging,^8,9^ and increasingly applications within the semiconductor industry for wafer metrology.^10^

Originally done with gases, recently there has been an increasing interest in HHG from solids.^4,11−13^ Solids are particularly interesting with regard to HHG as the generation process depends strongly on the electron dynamics, which can vary widely between solids. Various different solids have been used for HHG, including but not limited to semiconductors: Si,^14^ SiO,^15^ MgO,^16^ and ZnO;^13,17−19^ monolayers: graphene,^20,21^ MoS_2_,^22,23^ and WSe_2_;^24^ strongly correlated electron materials: VO_2_^25^ and NbO_2_;^26^ perovskites: MAPbBr_3_;^27^ and even metals: TiN.^28^ While this article focuses on HHG from solids, high-harmonics have also been generated from liquids^29,30^ and plasmas.^31,32^ This wide range of materials indicates the generality of the HHG process.

Moreover, the spatial structuring of solids allows for the fabrication of devices that enable further control of the HHG emission, examples include metasurfaces^33^ that can enhance HHG emission^34^ and allow for nonlinear beam steering.^35^ Furthermore, nanostructures can enable the shaping of EUV beams,^36^ and single nanocones^37^ and resonators^38^ provide means for miniaturizing HHG sources.

Lessons from research on atomic and molecular gas-phase HHG teach us that HHG can be controlled to great extent by adding additional laser pulses. Important examples include the emission control of odd and even harmonic orders in multicolor laser fields,^39−41^ controlling the harmonic emission intensity by aligning^42,43^ and orienting molecules,^44−47^ as well as controlling emission by photoexciting molecules.^48−54^

New developments in high-harmonic generation in solids provide an exciting prospect in enabling the study of microscopic electron dynamics,^23,25,55^ highly tunable (EUV) light sources,^37,56,57^ ultrafast all-optical signal modulation,^18−20^ and super-resolution imaging.^58^ Instrumental for all of these advancements is controlling high-harmonic generation. The literature on controlling gas HHG strongly suggests that all-optical emission control in solids is feasible. Recent works^17−20,22−25,59^ have shown strong modulation of high-harmonic generation, most notably strong suppression was observed. While the consistent observation of HHG suppression might be considered contrary to the dream of achieving extremely efficient solid HHG sources, tunable suppression does allow for advancements in applications such as those mentioned above. The power of suppression is very well exemplified in the recent demonstration of label-free super-resolution imaging in solids using harmonic deactivation microscopy (HADES),^58^ which has links to the successful fluorescence-based super-resolution technique stimulated emission depletion (STED). Here, the most crucial part is complete signal suppression and the ability to saturate this suppression. Additionally, the understanding of the suppression of high-harmonics may very well be the key to increasing high-harmonic generation for the purpose of an all-solid HHG source. Thus, for the development and optimization of high-harmonic modulation, it is important to obtain a more complete understanding of the underlying principles. In this Perspective, we will discuss the recent developments in the modulation of high-harmonicity from solids, provide an overview of the mechanism used to explain these recent observations, and provide an outlook into their main challenges.

As we aim to provide a general overview, we will mostly refrain from going into detail and discussing any material-specific properties. There are several effects that can play a dominant role in the HHG process in certain materials while being negligible or nonexistent in others. These effects include, for example, the presence of excitons,^60,61^ hot-carriers that are subject to study in investigations of photochemical processes probed by solid-state HHG,^27,62,63^ plasmonic effects,^64^ or even defect states.^18,65−67^ The impact of these effects on the HHG process is outside the scope of this Perspective. For the most part, our discussion assumes conventional semiconductor materials, except for the section on insulator-to-metal phase transition materials. These strongly correlated materials are deliberately included: They are particularly relevant in the context of HHG suppression because in recent studies the suppression observed in these materials was especially strong.^25,26^

## 2 High-Harmonic Generation in Solids

High-harmonic generation is a highly nonlinear strong field effect. Harmonics are generated from a high-intensity fundamental driver where the harmonic frequencies ω_*n*_ are multiples of the fundamental frequency ω_0_:
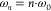
1

Normally only odd harmonics are generated due to the conservation of inversion symmetry.^11^ However, the effective generation of even harmonics can be achieved by using noninversion symmetric materials.^55,68−70^ Alternatively, multicolor fields consisting of, e.g., the fundamental frequency and its second harmonic have also been used to break the inversion symmetry and generate even harmonics.^14,71^ In solid intensities around GW/cm^2^^23,59^ to tens of TW/cm^2^^28^ are used for HHG, which is reached by the use of femtosecond pulsed lasers.

HHG is a strong-field effect where the electric field strength of the fundamental is in the same order of magnitude as the effective Coulomb force experienced by the valence electrons.^13^ As a result, HHG depends strongly on the fundamental electric field, as well as the material itself. This also means that an accurate description of HHG in solids requires a detailed description of both the fundamental field and the electronic structure of the material. For lower-intensity *n*-photon processes an intensity scaling of *I*_*n*_ ∝ *I*_0_^*n*^ is observed, which matches the predictions of perturbation theory.^72^ For higher harmonics, clear deviations for these ideal scalings are observed.^11,13^ This indicates that HHG can not simply be described using perturbation theory.

**Figure 1:**
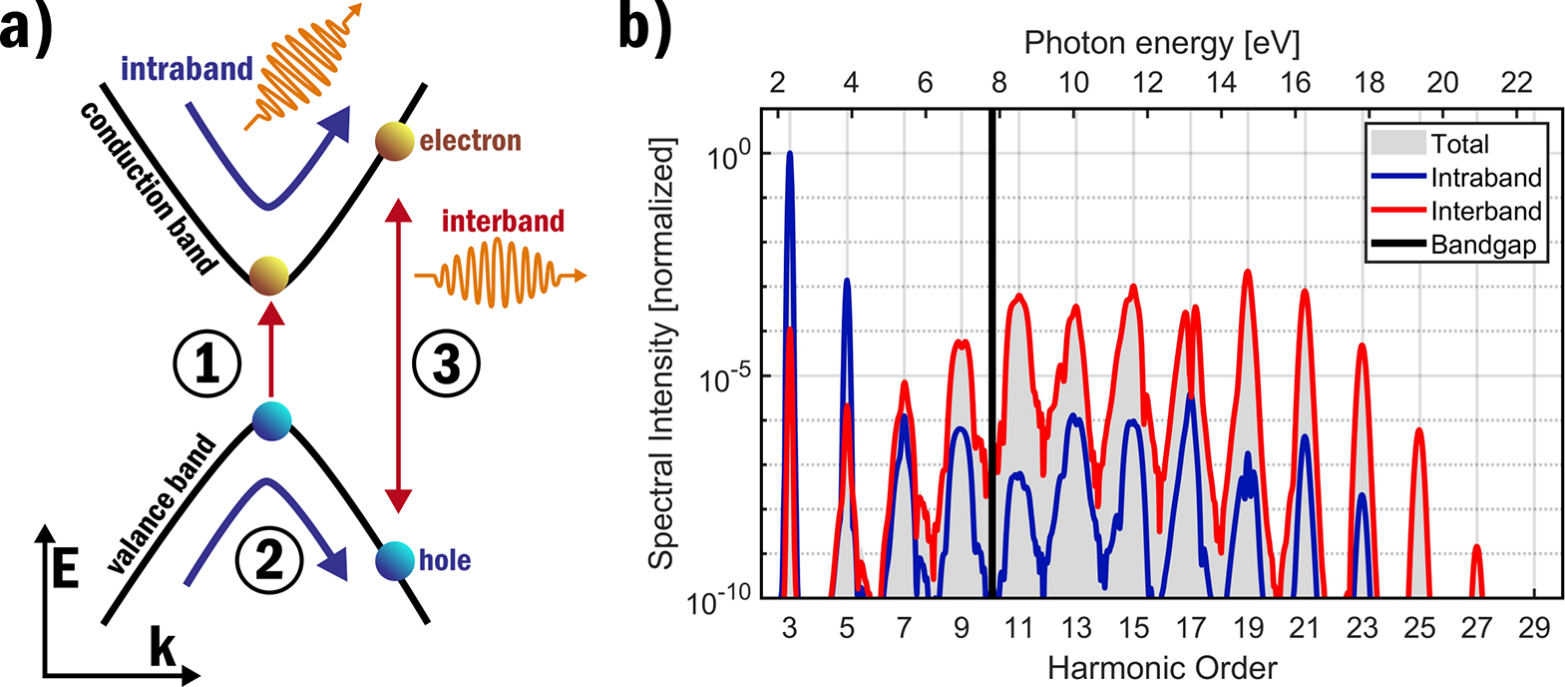
(a) Schematic of the three-step model for high-harmonic generation is shown in reciprocal space for a solid. In step **1**, the strong external electric field excites an electron–hole pair. In step **2**, after excitation, the electron–hole pair is accelerated by the strong electric field gaining kinetic energy. Acceleration along the nonparabolic bands of a solid generates the intraband current. In step **3**, the electron–hole pair recombines, which generates the interband current. The total high-harmonic signal is the combination of the intraband and interband contributions. (b) HHG spectrum is shown, which is simulated using a one-dimensional SBE simulation with two sinusoidal bands with a 7.8 eV bandgap and a 1 TW/cm^2^ generation pulse at 1600 nm. In blue (red) the intraband (interband) contributions to the HHG signal are shown.

The microscopic process underpinning high-harmonic generation is ultrafast and takes place on the attosecond time scale within an optical cycle of the fundamental. The harmonics are generated via the induced movement of charge carriers by a strong electric field. This effective current can be separated into the intraband and interband currents which are, respectively, related to transitions to different momenta within the same band and between different electronic bands, respectively. An introductory understanding of solid high-harmonic generation can be gained from the three-step model which is an adapted version of the three-step model used to explain gas high-harmonic generation.^4^ In the three-step model, a simplified two-band solid is considered with a single valence and a single conduction band that is illuminated by a strong electric field. In Figure 1, the three-step model for solids is schematically shown.

In the first step, the excitation of charge carriers is enabled by the strong field, deforming the Coulomb potential of the solid to such an extent that tunneling becomes possible. The excitation results in a coherent hole and electron in the valence and conduction bands. The excitation in a solid can occur throughout *k*-space, this is contrary to gas HHG where electron tunneling is confined close to *k* = 0 due to the conservation of momentum.^12^ In the second step, the excited electron and hole are accelerated by the strong electric field within their respective band, gaining kinetic energy. The movement of the electron and hole is impacted by the potential landscape of the solid. This step is distinctly different in gas HHG where tunneling electrons are accelerated through free space. During the acceleration of the electron–hole pair, the varying effective masses along the bands allow for the generation of the intraband current. The intraband current is not found in gas HHG, as the effective mass of charge carriers in the free space is constant. As the direction of the electric field changes within an optical cycle, the trajectory of the electron–hole pair can be such that they recombine, which is the third and final step. The recombination of the electron–hole pairs generates the interband current.

The significance of the intraband and interband contributions has been found to vary between harmonic orders.^73,74^ The distinction can be made that the intraband current generally contributes more to the lower-order below-bandgap harmonics. In contrast, the interband current generally plays a more significant role for the higher-order above-bandgap harmonics; this distinction can also be seen in the spectrum shown in Figure 1b. This separation can classically be understood: The intraband current arises from a nonlinear response to the electric field of the driving pulse that gives rise to higher-order frequency components in the current. This necessitates nonparabolic band structure or, equivalently, changes in effective mass as a function of carrier momentum. The changes in effective mass are usually small, thus giving rise to below-band gap harmonics. On the other hand, the interband current arises from electron–hole recollision between carriers in conduction and valence band in the classical picture and thus contributes energies that exceed the bandgap.

The semiclassical description as given above does have its limits, and to really obtain a predictive description of HHG in solids, the electron dynamics have to be considered quantum mechanically. This semiclassical model does however provide a very effective framework in which to understand the HHG process and the underlying suppression mechanisms.

A more quantitative description of the system, which accounts for the quantum mechanical nature, can be obtained using the semiconductor Bloch equations (SBE).^73,75,76^ Using the length gauge in the dipole approximation and the Bloch basis to describe the electronic states, the equation of motion (EOM) that is obtained for the density matrix elements with momentum **k** is given by^73^
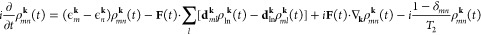
2**F**(*t*) here indicates the electric field, ϵ_*n*_^**k**^ is the energy level of the state, **d**_*nm*_^**k**^ is the dipole coupling between states *n* and *m*, *T*_2_ is the dephasing time, and δ_*mn*_ is the Dirac function. The diagonal elements of the density matrix (*m* = *n*) are the populations in the bands while the off-diagonal elements (*m* ≠ *n*) are the coherences between the bands. The right side of the EOM contains four distinct terms. The first term is a phase term and is dependent on the energy difference between the bands; the second term depends on the dipole couplings and describes transitions between the different bands; the third term depends on the derivative in k-space and describes the movement of carriers within a band; and the fourth term is a phenomenological damping term, which describes the dephasing between the carriers. Dephasing will be discussed in more detail in the section on excitation-induced dephasing. The interband and intraband current can be expressed as
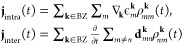
3BZ here refers to the Brillouin zone. We see that the intraband current is due to the movement of the population through a band, while the interband current is due to the time derivative of the coherent dipole coupling between bands. Important to note is that the intraband and interband currents are not entirely independent, as the EOM couples the population and coherence via the dipole coupling. The spectral intensity can be obtained by applying a Fourier transform to the total current:
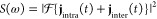
4The semiclassical interpretation as described by the three-step model can be derived from the SBE when considering a two-band system and applying the saddle-point approximation to the interband current.^4^

Commonly solid HHG is described in the reciprocal space; however, real space interpretations have also been used to gain additional insight into the generation process.^77,78^ We will explicitly discuss solid HHG in real space in the section on excitation-induced dephasing.

## 3 Time-Resolved High-Harmonic Generation

**Figure 2:**
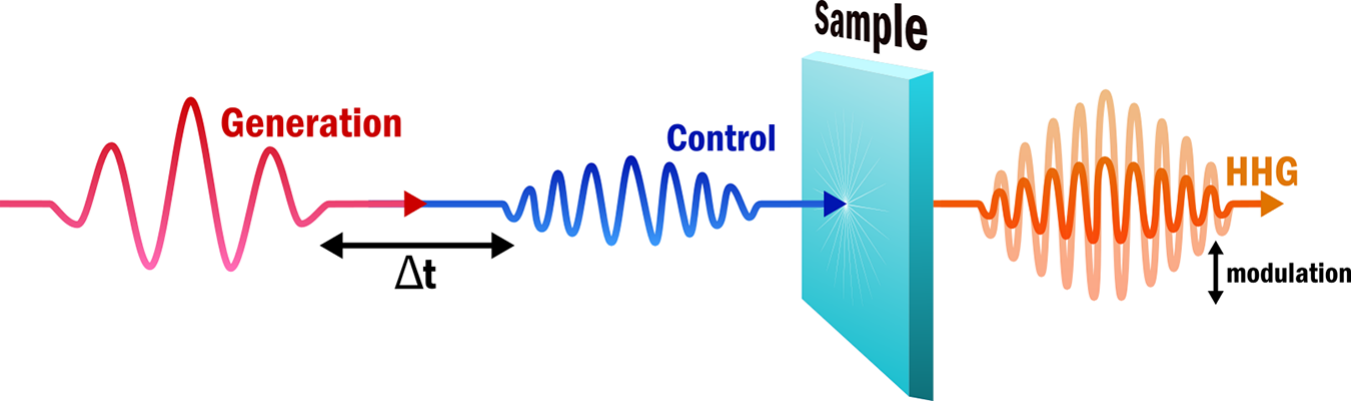
Schematic of the pump–probe system used to perform time-resolved high-harmonic generation. A high-intensity generation pulse is used to generate high-harmonics from a sample. An in space and time closely spaced control pulse is subsequently used to modulate the HHG. In the majority of measurements, the control pulse has a wavelength shorter than that of the generation pulse. By varying the delay time Δ*t* between the generation and control pulse time-resolved measurements are obtained. For positive delay times, the control pulse arrives before the generation pulse while for negative delay times, the control pulse arrives after the generation pulse.

Modulation of high-harmonic generation is achieved by introducing a control pulse, enabling pump–probe-style measurements, as shown in Figure 2. The control pulse is made to spatially overlap the generation pulse such that it can affect the HHG process. By varying the delay time between the generation and control pulses time-resolved spectroscopy can be performed. Positive time delays refer to the situation where the control pulse arrives before the generation pulse while negative time delays refer to the situation where the generation pulse arrives first. The intensity used for the control pulse varies widely but is often lower than that of the generation pulse so as to not generate harmonics itself or damage the sample. Different implementations of this same measurement scheme have been used in nearly all recent works which have shown modulation of HHG in solids.^17−20,22−25^ An overview of these recent works with the materials and wavelengths used can be found in Table 1.

**Table 1:** Overview of Recent Works Showing Suppression of HHG in Various Materials^a^

mechanism(s)	material	λ_gen_ (nm)	λ_con_ (nm)	HO	suppression	ref
state blocking	ZnO	3500	400	7, 11, 13	90%^(11,13)^	(17)
state blocking	MoS_2_	1560–2385	400	3, 4, 5	95%^(4)^	(22)
state blocking	graphene	1350	400, 800	3	90%^(3)^	(20)
EID	ZnO	2350	400, 800	5, 7	95%^(5)^	(18)
EID	MoS_2_	5000	660	5–16	85%^(16)^	(23)
EID (state blocking^b^)	WSe_2_	4770	760	5, 7, 9, 10, 12	90%^(9,10,12)^	(24)
EID	MAPbBr^c^	1440–2320	400	3, 5	75%^(3)^	(27)
IMT	VO_2_	7000, 10000	1500	5, 7, 9	99%^(3,5)^^d^	(25)
IMT	NbO_2_	1800, 2160	400	3, 5	99%^(3,5)^^d^	(26)
field modulation	ZnO	3500	1300	9, 11	80%^(9)^	(19)

a The mechanisms indicate the suppression mechanisms discussed in these works, these being state blocking, excitation-induced dephasing (EID), insulator-to-metal phase transitions (IMT), and field modulation. λ_gen_ and λ_con_, respectively, indicate the generation and control wavelength used. HO indicates the harmonic orders that have been shown from these experiments. Suppression indicates the approximate maximum intensity suppression reported for the harmonic(s) denoted between the brackets. In a number of works the suppression is explicitly stated while for others the maximum suppression has been evaluated based on the figures presented. It is of note that the majority of works presented here have not explicitly sought after optimization of this suppression.

b State blocking is explicitly discussed, however, is determined not to be the main cause of suppression.

c MAPbBr: methylammonium lead bromide.

d In these measurements the harmonic intensity dropped below the detection threshold.

**Figure 3:**
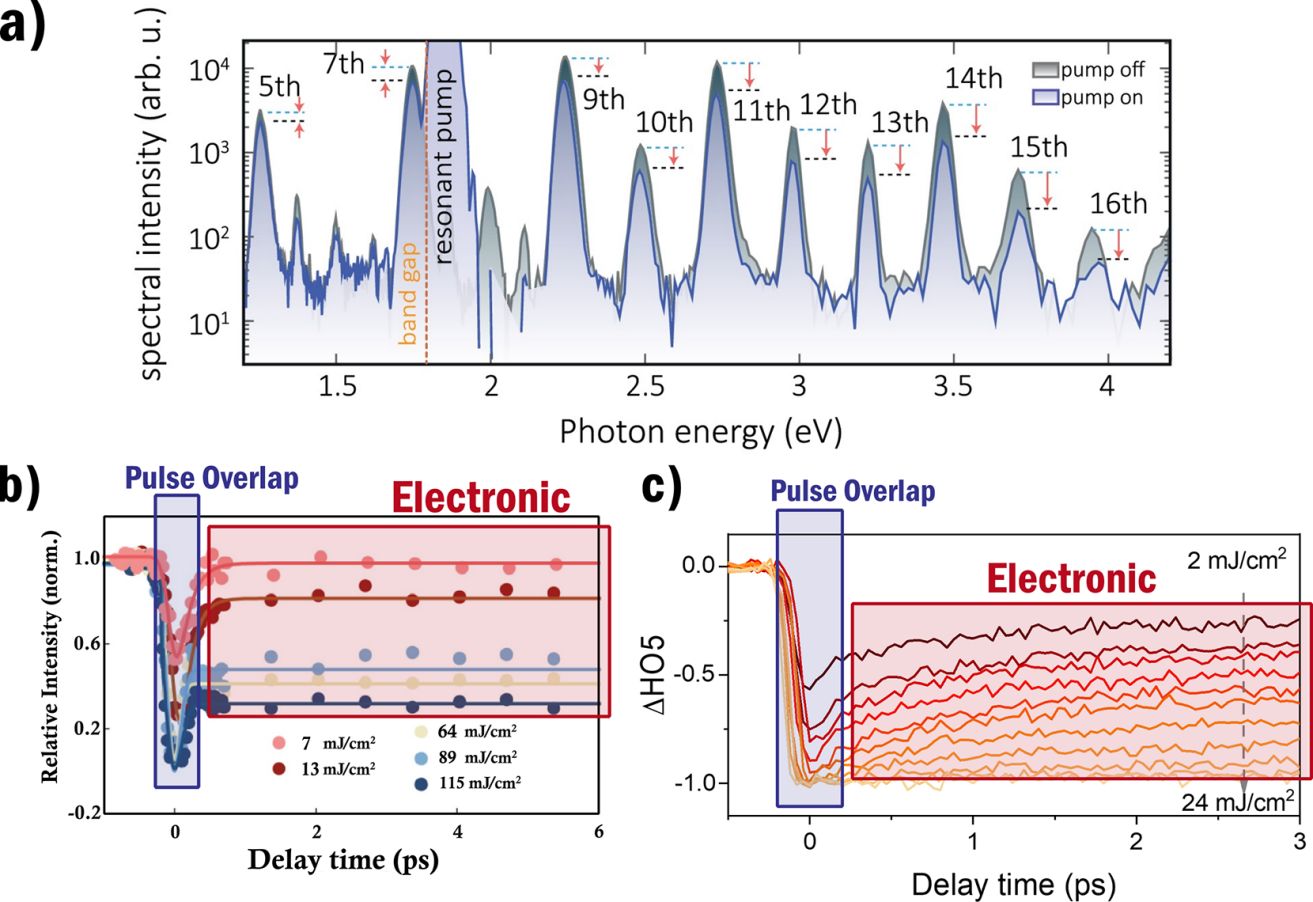
In (a) the harmonic spectra by ref (23) from MoS_2_ using 5000 nm as generation wavelength and 660 nm as control wavelength. The two different spectra show the control pulse being, respectively, on and off, the delay time is 1 ps. The arrows indicate the decrease in intensity for each of the harmonics. In (b) the suppression curve measured by ref (18) is shown for the fifth harmonic from ZnO using 2350 and 800 nm, respectively, as generation and control wavelengths. In (c) the suppression curve measured by ref (26) is shown for the fifth harmonic from NbO_2_ using 2000 and 400 nm, respectively, as generation and control wavelengths. We note that the curves in (b) and (c) show very similar behavior although they are measured from vastly different materials. A sharp drop in HHG yield is observed when the generation and control pulse overlap, while for longer delay times we see a gradual recovery of the intensity. In both (b) and (c), the overlap and electronic recovery region are indicated explicitly. Increased suppression is found for increasing control intensity. (a) is reprinted with permission from ref (23). Copyright 2022 Optica Publishing Group. (b) is reprinted and adapted with permission from ref (18). Copyright 2022 Optica Publishing Group. (c) is reprinted and adapted with permission under the Creative Commons CC-BY 4.0 from ref (26).

Although various materials have been studied using significantly different measurement conditions, the results have shown some rather noticeable consistencies, the most important of these being strong suppression. Figure 3a shows the HHG spectra measured by ref (23) from ZnO for a 1 ps pulse delay where increasing suppression is observed for the harmonic orders. In Figure 3b the suppression of the fifth harmonic from ZnO measured by ref (18) is shown as a function of delay time. Per comparison, Figure 3c shows a similar figure but now for the fifth harmonic measured from NbO_2_ by ref (26). The suppression curves shown in Figure 3b,c are exemplars for HHG suppression observed in solids.

For negative delay times, no change in the harmonics is observed as the control pulse arrives after the generation process. When the control pulse starts to overlap with the generation pulse, a strong suppression is observed. During pulse overlap, the control pulse can directly affect the carrier dynamics during HHG. Significant suppression is also observable outside the direct overlap region for positive delay times, where for increasing delay time the suppression gradually recovers up to the unsuppressed HHG yield. For these delay times, the control pulse can not directly affect the HHG process but instead does this via the excitation of charge carriers. The presence of an initial carrier population generated by the control pulse is what causes the suppression. The carrier population will over time be affected by relaxation and recombination, which bring the excited charge carriers back to their ground state; this is directly observed in the recovery of harmonic yield for longer delay times. Increased suppression is found for both increasing control intensity and increasing harmonic order. The fact that the suppression increases with harmonic order strongly suggests that the underlying suppression mechanisms predominantly affect the interband current.

## 4 Mechanisms of Suppression of High-Harmonic Generation

So far we have described the common observation of HHG suppression and its significant features; in the next section, we will discuss the specific microscopic mechanisms that can explain these observations. Four main possible mechanisms were identified, these being state blocking, excitation-induced dephasing (EID), insulator-to-metal phase transitions (IMT), and field modulation. Table 1 shows which of these mechanisms have been discussed in recent works in which suppression was observed. In the following sections we will discuss these mechanisms individually.

### 4.1 State Blocking

A number of works^17,20,22,24^ have looked into the effects of state blocking in order to explain the observed suppression. The idea here is that the control pulse generates an initial carrier population that occupies some of the excited states. The occupation of these excited states prevents the excitation of coherent electron–hole pairs to these states by the generation pulse. Similarly, the lack of electrons in the valence band will also inhibit excitation; this is referred to as ground state depletion. When the excitation by the generation pulse is prevented, the HHG is suppressed.

The effects of state blocking and ground state depletion have been observed in a variety of different systems^79−82^ that motivates the investigation of this mechanism in the context of HHG suppression.

**Figure 4:**
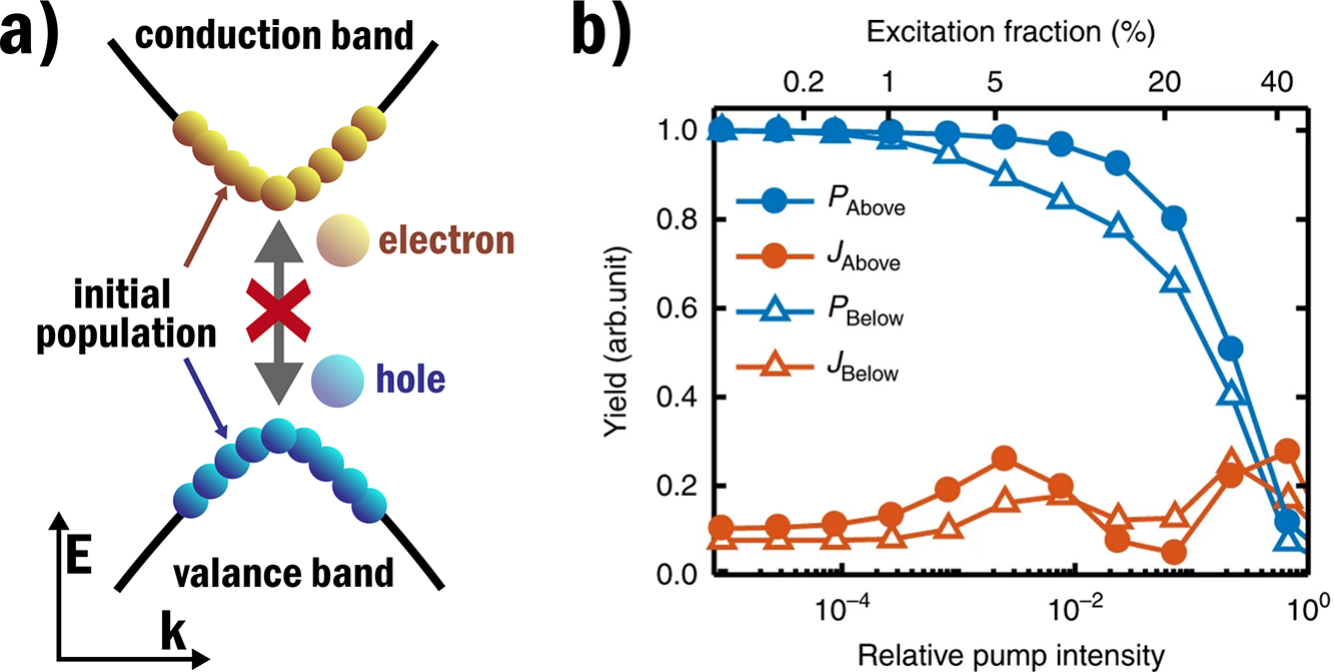
In (a) the effect of state blocking is schematically shown where the generation of new electron–hole pairs is inhibited by the presence of an initial carrier population. In (b) SBE simulation results for the harmonic yield generated by a 3500 nm generation pulse with a 400 nm control pulse in ZnO, by ref (17), *P* and *J*, respectively, indicate the interband and intraband contributions and the yield is shown separately for the above and below bandgap harmonics. The yield is shown as a function of relative control intensity, as well as the corresponding excitation fraction. The corresponding measurements in ref (17) showed near complete suppression, so for these simulations to match, excitation fractions over 40% are required. (b) is reprinted and adapted with permission under the Creative Commons CC-BY 4.0 from ref (17).

The impact of state blocking has been investigated using simulations based on SBE. These SBE simulations make use of second-quantization to describe the electron dynamics, which enables the simulation of state blocking. Different SBE simulations^17,24^ show that the effects of state blocking are likely negligible for realistic parameters. In Figure 4, the results of an SBE simulation of HHG in ZnO are shown. In the corresponding experiments,^17^ near-complete suppression of the harmonics was observed that would require excitation fractions above 40% to be consistent with these simulations. Realistic excitation fractions in semiconductors are considered to be only a few percent, as above this dielectric breakdown occurs, which results in material damage. The big discrepancy between simulations and experiments is a strong indication that state blocking by itself can not solely account for the significant observed suppression of HHG.

### 4.2 Excitation-Induced Dephasing

**Figure 5:**
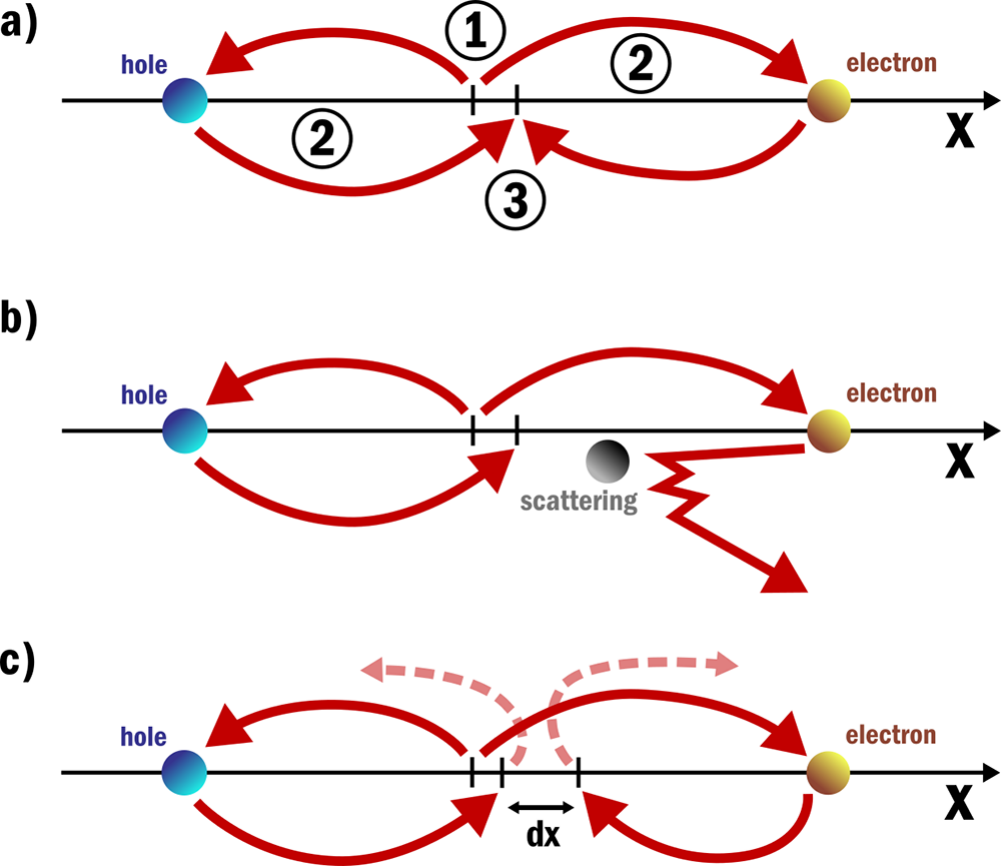
In (a) the three-step model is schematically shown in real space. In step **1** an electron–hole pair is generated, in step **2** the charge carriers are accelerated, and in step **3** the electron and hole recombine. In (b) a scattering event of one of the charge carriers during the propagation is included. The scattering event causes the electron and hole to lose spatial coherence and prevents their coherent recombination. The scattering shown here can be carrier–carrier scattering, carrier-phonon scattering, or scattering of defects all of which will result in the loss of coherence. In (c) the electron and hole recombination is inhibited by an induced spatial displacement d*x*. This spatial displacement can be induced by having an additional electric field present during the acceleration of the electron–hole pair.

Coherence between the generated electron–hole pair is essential for effective recombination, resulting in the interband current. To effectively illustrate this, we can consider the semiclassical three-step model in real space, as is shown in Figure 5a. After excitation, the electron and hole are spatially separated, as they are driven by the electric field. If during propagation one of the carriers scatters, as shown in Figure 5b, part of its momentum and/or energy is transferred. As a result of this scattering, the electron–hole pair will lose spatial coherence and will not coherently recombine. Figure 5c shows an alternative manner in which recombination can be inhibited; this figure will be discussed in more detail in the section on field modulation. The loss of spatial coherence is also referred to as dephasing. Dephasing can be caused by all different scattering events, including carrier–carrier scattering, carrier–phonon scattering, and scattering of defects.

The importance of dephasing has consistently been confirmed by the modeling of HHG. In models, the dephasing effectively functions as a damping term that stops the buildup of the coherent population over multiple optical cycles. As the particle density of solids greatly exceeds that of gases, dephasing plays a significantly greater role.

In order to match simulations of HHG from solids with experiments, short dephasing times of only a few femtoseconds have to be used.^73,74^ This is considerably lower than the dephasing time found with photon-echo measurements, where dephasing times are closer to tens of femtoseconds.^83^ By including the macroscopic propagation it is possible to explain the discrepancy between dephasing times.^84^ This propagation-induced dephasing effect does however require sample thicknesses of at least a few micrometers and thus can not explain the short dephasing times of thin and monolayer samples. More recently, by considering dephasing in real space the short dephasing times were attributed to recombination events of carriers that experience large spatial separation.^77^ We also note that for HHG the carriers are accelerated to much higher momenta than those in photon-echo measurements. Therefore, we see the significant discrepancy in dephasing times as a strong indication that the dephasing time is strongly dependent on the carrier momentum.

The excitation of carriers by the control pulse can cause an alteration of the dephasing time by increasing the likelihood of scattering. This is either directly by the presence of more excited carriers or mediated via phonon coupling. A reasonably lowered dephasing time can account for the significant suppression observed in experiments.^23,24^ Moreover, the observation of strong harmonic dependence when it comes to suppression is a convincing sign that dephasing dominates high-harmonic suppression. This can intuitively be understood, as higher harmonics require longer acceleration times and thus will be affected more when the dephasing rate is increased.^23^

Control over the dephasing rate in solids will thus enable major control over high-harmonic generation in a material. Interestingly, a reduction of the dephasing rate should allow for a significant increase in the high-harmonic yield.

The difficulty with dephasing is that it results from carrier scattering, which is intrinsically a multielectron effect, as well as carrier-phonon scattering, which requires an accurate description of electron–phonon coupling. Photon-echo experiments^83^ have measured photocarrier-density dependent dephasing times that are linked to carrier scattering, but only at low excitation intensities, for single-photon absorption at the Γ point, all conditions that are not fulfilled in HHG. Current state-of-the-art simulations either make use of SBE or density functional theory (DFT). In SBE simulation dephasing is added via a phenomenological damping term to the EOM, as is also shown in eq 2.^73^*T*_2_ is the dephasing time, while *T*_1_ refers to the recombination time. Conventionally in SBE simulations, *T*_2_ is chosen to accomplish good agreement between simulation results and measurements.^73,85^ This means that SBE simulations that do not explicitly calculate *T*_2_ have no predictive power when it comes to the dephasing time. Time-dependent DFT simulations have been applied to solid HHG^86^ and allow for the inclusion of some scattering effects depending on the choice of the exchange-correlation functional. The complexity of DFT simulations, however, can make gaining physical intuition challenging, and the multielectron nature of *T*_2_ makes it particularly difficult to grasp in DFT. Moreover, some dephasing effects are not easily implemented in DFT simulations, such as the contribution of material defects.

New advancements in modeling and understanding the scattering in solids as causing dephasing in high-harmonic generation will be key to the advancement of all-optical high-harmonic modulation.

### 4.3 Insulator-to-Metal Phase Transitions

Both state blocking and excitation-induced dephasing focus on carrier interactions and do not consider significant changes in electronic potential in the material. For most conventional materials carrier excitation has a limited effect on the electronic structure, with effects such as bandgap renormalization being small.^87^

Contrary to this are strongly correlated materials (SCMs) which can undergo phase transition under carrier excitation.^88−91^ Significant changes to the electronic structure will have a major impact on the carrier dynamic and as a consequence also on the HHG emission. In this section, we will focus on a particular class of SCMs, where the insulator-to-metal phase transition (IMT) has been found, and also studied via HHG.^25,26^

**Figure 6:**
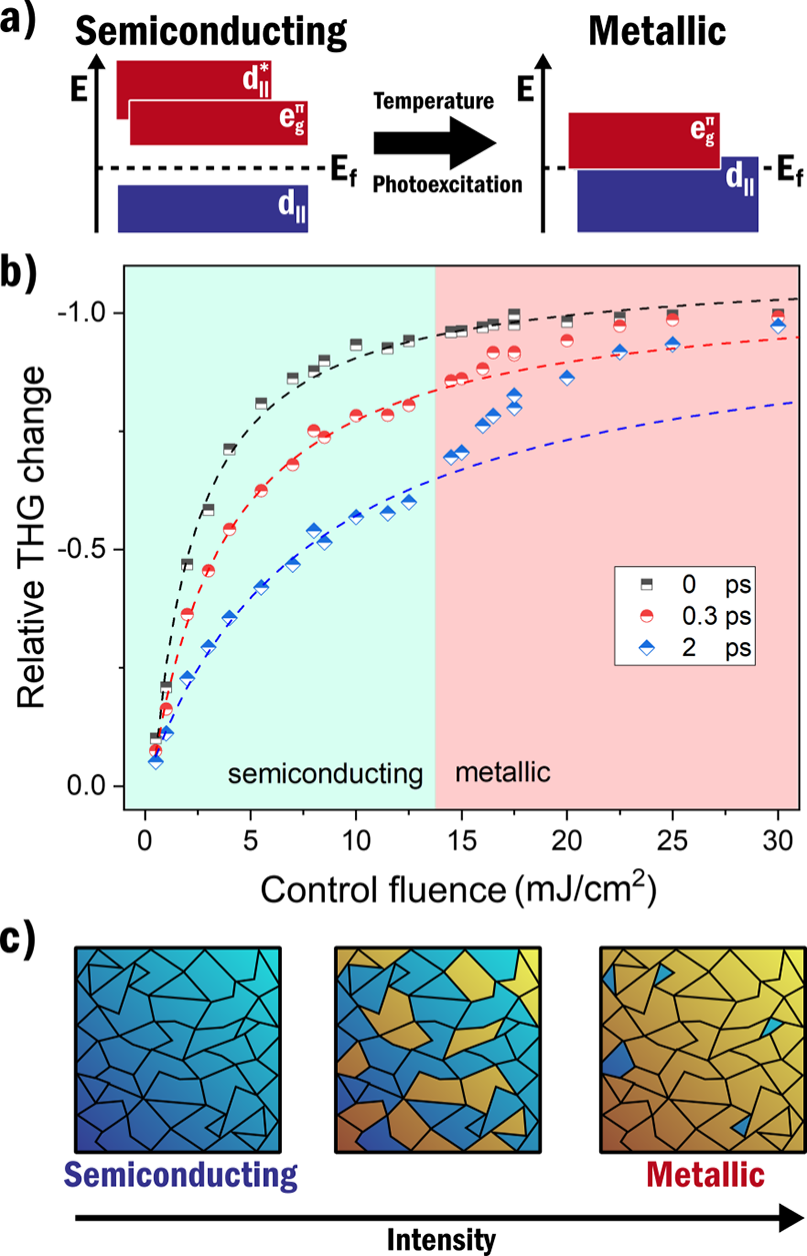
(a) Restructuring of the energy states around the bandgap of NbO_2_ when it undergoes the insulator-to-metal phase transition. This phase transition can be initialized by temperature (around 1080 K) or by photoexcitation. In the semiconducting state, NbO_2_ has a bandgap of around 0.7–1.1 eV. In (b) the suppression of the third harmonic from NbO_2_ is shown for increasing control intensity for three different pulse delays measured by ref (26). 1800 nm generation pulses were used in combination with 400 nm control pulses, and the measurements were performed at room temperature. A clear deviation from the exponential suppression is observed at around 13.5 mJ/cm^2^, indicating the material phase transition. In (c) the spatial states of NbO_2_ are shown for increased illumination intensity. Segments of semiconducting and metallic states can exist within a NbO_2_ sample at the same time. To study individual segments high-resolution spatially resolved measurements are required. (b) is reprinted and adapted with permission under the Creative Commons CC-BY 4.0 from ref (26).

IMT in SCMs refers to the material switch from the insulating or semiconducting phase to the metallic phase under certain external triggers, such as temperature or photoexcitation.^89−91^ As an example, Figure 6a schematically shows the IMT in NbO_2_ where the merging of the separated orbitals around the Fermi level results in the collapse of the bandgap.^89^ Due to their unique electronic properties, SCMs have been of significant interest to the development of new novel devices. For example, IMT materials are a key enabler of Mott memristors which in turn pave the way for neuromorphic computing.^92,93^ While IMT in SCMs has been studied over the past decade, a number of relevant open questions remain. For example, how to distinguish between the competing electron–electron and electron–lattice interactions in the IMT.

Due to its highly nonlinear nature, HHG is extremely sensitive to the IMT, as HHG emission can be greatly altered by any subtle change in electronic or lattice structures, let alone the bandgap collapse. It is possible to directly observe the IMT using time-resolved HHG. Figure 6b shows the IMT in NbO_2_ at a control fluence of 12 mJ/cm^2^ via the clear deviation from a saturation model.^26^ The saturation model considers the suppression due to the lowered dephasing time in the semiconducting state. The deviation corresponds to a sudden increased suppression of HHG and is assigned to photoinduced IMT.

To explain the greatly reduced HHG efficiency in the metallic phase two main effects can be considered: first, a higher density of free carriers in the metallic phase results in a significantly reduced dephasing time; second, the bandgap collapse results in carriers movement closer to that of free carriers.^28^ Free carriers miss the nonlinear response to the driving field required for generating high-harmonics. Both effects together can result in a very strong, practically complete signal suppression, as shown in Figure 6b.

Besides all-optical control of HHG, this very strong signal suppression enables HHG as an ultrasensitive probe for ultrafast nanoscopy in SCMs. As the HHG process takes place within an optical cycle, it becomes possible to resolve the phase transitions with very high temporal resolution of only a few femtoseconds and possible subfemtosecond. This will enable the identification of electronic contributions in the IMT.

Except for the temporal properties of IMT, spatial information could also be accessed using HHG imaging. As shown in Figure 6c, the IMT will not occur throughout the whole material at the same time. Spatial imaging of the IMT will benefit greatly from the aforementioned super-resolution imaging techniques which make use of the HHG modulation and have already been demonstrated to work for NbO_2_.^58^

Optical modulation of HHG in solids is promising in simultaneously providing the temporal and spatial information on IMT, which not only benefits a comprehensive understanding of IMTs but can also guide the device design with SCMs.

### 4.4 Field Modulation

So far, we have discussed mechanisms where HHG was affected via carrier excitation of the control pulse. When there is overlap between the generation and control pulse, the field of the control pulse can affect the electron dynamics of the HHG process directly. During overlap, we can consider the control pulse as a modulation of the generation field.

Shaped multicolor fields have been demonstrated to allow for an increased HHG yield, an increased cutoff frequency, and divergence control in gases.^40,41,94,95^ In these cases, the field is shaped such that more of the excited charge carriers can coherently recombine by tuning the excitation rate and trajectories. While these works focus on increasing the HHG yield, the same principle can be used to achieve suppression by instead inhibiting coherent recombination. We can imagine this suppression in real space by considering that the control field induces a spatial displacement between carriers, as shown in Figure 5c. The control field can affect the trajectories such that the induced spatial displacement inhibits recombination and, as a result, suppresses the harmonics. Alternatively viewed, the control field can modulate the carrier movement through reciprocal space, which allows it to impact the intraband current. Additionally, for strong enough control fields, the time at which significant excitation occurs can also be altered.

**Figure 7:**
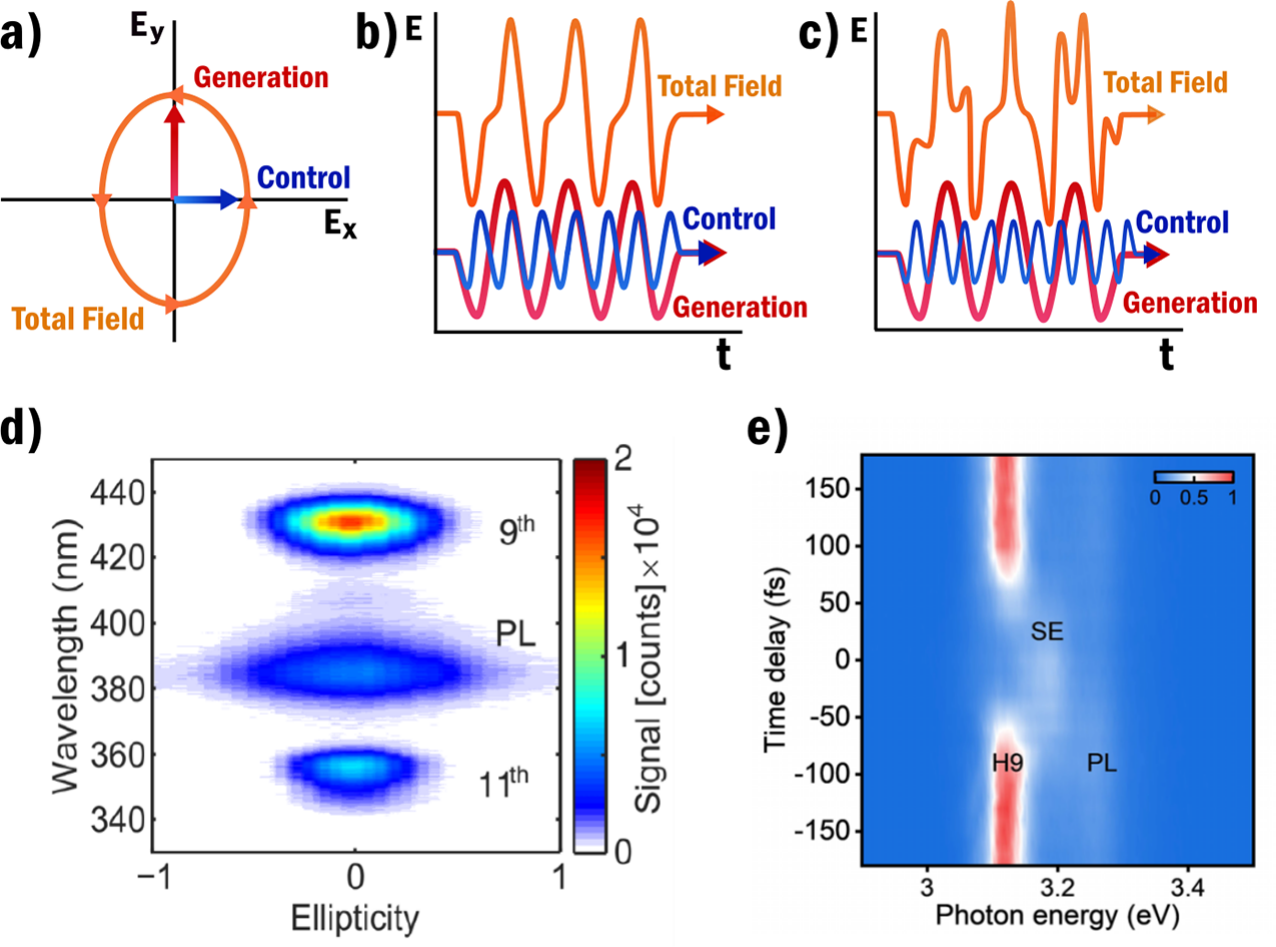
In (a), (b), and (c) the different frequency relations between the generation ω_*g*_ and control ω_*c*_ pulse are shown. In (a) the generation and control frequencies are degenerate, which means that substantial field changes can only be accomplished when the fields are out-of-phase and orthogonal such that elliptical polarization can be achieved. In (b) the control frequency is an integer multiple of the generation frequency which results in equal modulation of every optical cycle. In (c) the control frequency is not an integer multiple of the generation frequency such that every optical cycle is modulated differently. In (b) and (c) the total field curve has been offset to better show its shape. In (d) ZnO HHG results from ref (97) are shown for different generation ellipticity. The ellipticity of −1 and 1 corresponds to the left- and right-handed circular polarization, while 0 corresponds to linear polarization. PL indicates the photoluminescence. In (e) ZnO HHG results from ref (19) are shown for 3500 nm generation and 1300 nm control wavelengths. As the control wavelength far exceeds the bandgap of ZnO (around 368 nm^18,98^) suppression can only be observed in the region of pulse overlap. PL indicates the photoluminescence and SE indicates some stimulated emission signal. (d) is reprinted and adapted with permission under the Creative Commons CC-BY 4.0 from ref (97). (e) is reprinted with permission from ref (19). Copyright 2023 American Physical Society.

Depending on the wavelength ratio between the generation and control pulse, various modulations of the electric field are possible. One way of modulating the generation pulse is by having it become elliptically polarized, this is equivalent to adding a degenerate out-of-phase orthogonal component to the generation field, as is also shown in Figure 7a. In practice, elliptical polarization is achieved by making use of quarter-waveplates. For various materials, near-complete HHG suppression is observed when ellipticity is increased,^21,96,97^ an example of which is shown in Figure 7d where also a clear harmonic dependence is observed.

To effectively control the field shape during an optical cycle, nondegenerate wavelengths have to be used. To modulate all optical cycles equally, the control frequency has to be an integer multiple of the generation frequency, as shown in Figure 7b, if this is not the case all optical cycles are modulated differently, as shown in Figure 7c. For the aforementioned multicolor HHG process, second and third harmonic generations are used to allow for consistent modulation of the optical cycles. For efficient suppression, this consistent modulation is not necessary, as is shown by the results in Figure 7e where near complete suppression is observed by using a noncommensurate control pulse. Note that the suppression is only observed in the region of overlap as the control pulse in this experiment is far enough below the bandgap such that it does not excite carriers. It is also visible in Figure 7e that at the temporal overlap of HHG generation pulse and control pulse, there is an increased stimulated emission. This is linked to increased electron–electron impact excitation, which correlates with decreased HHG efficiency. This correlation could suggest that conditions that promote impact excitation may be the most advantageous for deactivating HHG, which presents a further avenue for future research.

Modulation and suppression have mostly been found to be the most significant during overlap; as a result, applications utilizing HHG suppression will likely rely on processes governed by the effect of field modulation. As there is no requirement for exciting charge carriers, there is a lessened importance of the material and an increased importance of the electric field. The loosening of this requirement also allows the use of higher wavelength and lower intensity control pulses for achieving significant suppression.

As the modulation in the system is caused just by the electric field, simulations of these systems are much more feasible compared to those considering dephasing, where scattering interactions have to be considered. An exciting challenge lies in the optimization of the control pulse for maximum modulation of high-harmonic generation, certainly considering the great number of possible parameters to optimize.

## 5 Conclusion and Outlook

In this Perspective, the microscopic mechanisms underlying the suppression of HHG in solids have been discussed. Four main mechanisms were identified, these being state blocking, excitation-induced dephasing, insulator-to-metal phase transitions, and field modulation.

Simulations of state blocking indicated only a minor contribution to the suppression of HHG in conventional semiconductors, which indicates that state blocking by itself can not account for the significant suppression observed in recent measurements.

Excitation-induced dephasing can account for the significant suppression observed, as well as the further temporal dynamics observed in experiments. The scattering underlying dephasing makes it difficult to model and exceeds the limits of current state-of-the-art simulations. An interesting challenge lies in enabling predictive modeling of the dephasing, which, in turn, would enable the precise study of the underlying electron and phonon dynamics.

For IMT materials, a significantly increased HHG suppression occurs due to the bandgap collapse. Due to the possibility for high temporal and spatial resolution, HHG provides an exciting platform for resolving the material phase transitions, which will greatly benefit the development of SCMs devices.

The most significant suppression is consistently found during pulse overlap, where the control field effectively modulates the generation field. The significance of the observed suppression makes this mechanism especially relevant for applications such as all-optical signal modulation and super-resolution imaging. The significant degrees of freedom with regard to generation and control pulse combinations open the door for precise field optimization to obtain a very controlled modulation of HHG.

Improving our understanding of these microscopic mechanisms underpinning the HHG process will be key to achieving complete optical control of the HHG process. Such complete all-optical control has numerous applications. We have elaborated on the potential for label-free super-resolution microscopy via deactivated high-harmonic generation in solids. Additionally, by controlling the carrier excitation during the HHG process, subdiffraction-controlled chemistry on the nanoscale may become possible. This seems particularly feasible for field modulation, where experience from gas HHG has shown that the excitation step of HHG can be controlled effectively.^40,41^ Finally, nonlinear solid light sources are explored in many fields, for example, via nonlinear dielectric metasurfaces^33^ that offer great deals of beam control via nanoscale engineering of structures. Optical control of HHG adds another control knob that enables femtosecond switching of these metasurfaces. Consequently, we are convinced that optical control of HHG has numerous applications in the future, as it represents a fully controllable femtosecond, possibly even subfemtosecond, all-optical switch.

## References

[ref1] Corkum P. B.; Krausz F. Attosecond science. Nat. Phys. 2007, 3, 381–387. 10.1038/nphys620.

[ref2] Calegari F.; Sansone G.; Stagira S.; Vozzi C.; Nisoli M. Advances in attosecond science. Journal of Physics B: Atomic, Molecular and Optical Physics 2016, 49, 062001 10.1088/0953-4075/49/6/062001.

[ref3] Li J.; Lu J.; Chew A.; Han S.; Li J.; Wu Y.; Wang H.; Ghimire S.; Chang Z. Attosecond science based on high harmonic generation from gases and solids. Nat. Commun. 2020, 11, 2748 10.1038/s41467-020-16480-6. 32488005 PMC7265550

[ref4] Vampa G.; Brabec T. Merge of high harmonic generation from gases and solids and its implications for attosecond science. Journal of Physics B: Atomic, Molecular and Optical Physics 2017, 50, 083001 10.1088/1361-6455/aa528d.

[ref5] Kraus P. M.; Zürch M.; Cushing S. K.; Neumark D. M.; Leone S. R. The ultrafast X-ray spectroscopic revolution in chemical dynamics. Nature Reviews Chemistry 2018, 2, 82 10.1038/s41570-018-0008-8.

[ref6] Kraus P. M.; Wörner H. J. Perspectives of attosecond spectroscopy for the understanding of fundamental electron correlations. Angew. Chem., Int. Ed. 2018, 57, 5228–5247. 10.1002/anie.201702759. 29624808

[ref7] Wörner H. J.; Arrell C. A.; Banerji N.; Cannizzo A.; Chergui M.; Das A. K.; Hamm P.; Keller U.; Kraus P. M.; Liberatore E.; et al. Charge migration and charge transfer in molecular systems. Structural dynamics 2017, 4, 061508 10.1063/1.4996505. 29333473 PMC5745195

[ref8] Jansen G. S. M.; de Beurs A.; Liu X.; Eikema K. S. E.; Witte S. Diffractive shear interferometry for extreme ultraviolet high-resolution lensless imaging. Opt. Express 2018, 26, 12479–12489. 10.1364/OE.26.012479. 29801285

[ref9] Loetgering L.; Liu X.; De Beurs A. C.; Du M.; Kuijper G.; Eikema K. S.; Witte S. Tailoring spatial entropy in extreme ultraviolet focused beams for multispectral ptychography. Optica 2021, 8, 130–138. 10.1364/OPTICA.410007.

[ref10] Porter C.; Coenen T.; Geypen N.; Scholz S.; van Rijswijk L.; Nienhuys H.-K.; Ploegmakers J.; Reinink J.; Cramer H.; van Laarhoven R.; et al. Soft X-ray: novel metrology for 3D profilometry and device pitch overlay. Metrology, Inspection, and Process Control 2023, XXXVII, 412–420. 10.1117/12.2658495.

[ref11] Goulielmakis E.; Brabec T. High harmonic generation in condensed matter. Nat. Photonics 2022, 16, 411–421. 10.1038/s41566-022-00988-y.

[ref12] Huttner U.; Schuh K.; Moloney J. V.; Koch S. W. Similarities and differences between high-harmonic generation in atoms and solids. Journal of the Optical Society of America B 2016, 33, C22 10.1364/JOSAB.33.000C22.

[ref13] Ghimire S.; Dichiara A. D.; Sistrunk E.; Agostini P.; Dimauro L. F.; Reis D. A. Observation of high-order harmonic generation in a bulk crystal. Nat. Phys. 2011, 7, 138–141. 10.1038/nphys1847.

[ref14] Vampa G.; Hammond T. J.; Thiré N.; Schmidt B. E.; Légaré F.; Klug D. D.; Corkum P. B. Generation of high harmonics from silicon. arXiv:1605.06345 [physics.optics] 2016, na 10.48550/arXiv.1605.06345.

[ref15] Luu T. T.; Garg M.; Kruchinin S. Y.; Moulet A.; Hassan M. T.; Goulielmakis E. Extreme ultraviolet high-harmonic spectroscopy of solids. Nature 2015, 521, 498–502. 10.1038/nature14456. 26017451

[ref16] You Y. S.; Reis D. A.; Ghimire S. Anisotropic high-harmonic generation in bulk crystals. Nat. Phys. 2017, 13, 345–349. 10.1038/nphys3955.

[ref17] Wang Z.; Park H.; Lai Y. H.; Xu J.; Blaga C. I.; Yang F.; Agostini P.; DiMauro L. F. The roles of photo-carrier doping and driving wavelength in high harmonic generation from a semiconductor. Nat. Commun. 2017, 8, 1686 10.1038/s41467-017-01899-1. 29162818 PMC5698516

[ref18] Xu S.; Zhang H.; Yu J.; Han Y.; Wang Z.; Hu J. Ultrafast modulation of a high harmonic generation in a bulk ZnO single crystal. Opt. Express 2022, 30, 41350 10.1364/OE.462638. 36366615

[ref19] Wang Y.; Liu Y.; Jiang P.; Gao Y.; Yang H.; Peng L.-Y.; Gong Q.; Wu C. Optical switch of electron-hole and electron-electron collisions in semiconductors. Phys. Rev. B 2023, 107, L161301 10.1103/PhysRevB.107.L161301.

[ref20] Cheng Y.; Hong H.; Zhao H.; Wu C.; Pan Y.; Liu C.; Zuo Y.; Zhang Z.; Xie J.; Wang J.; Yu D.; Ye Y.; Meng S.; Liu K. Ultrafast optical modulation of harmonic generation in two-dimensional materials. Nano Lett. 2020, 20, 8053–8058. 10.1021/acs.nanolett.0c02972. 33112622

[ref21] Yoshikawa N.; Tamaya T.; Tanaka K. Optics: High-harmonic generation in graphene enhanced by elliptically polarized light excitation. Science 2017, 356, 736–738. 10.1126/science.aam8861. 28522530

[ref22] Wang Y.; Iyikanat F.; Bai X.; Hu X.; Das S.; Dai Y.; Zhang Y.; Du L.; Li S.; Lipsanen H.; Abajo F. J. G. D.; Sun Z. Optical Control of High-Harmonic Generation at the Atomic Thickness. Nano Lett. 2022, 22, 8455–8462. 10.1021/acs.nanolett.2c02711. 36305718 PMC9650768

[ref23] Heide C.; Kobayashi Y.; Johnson A. C.; Liu F.; Heinz T. F.; Reis D. A.; Ghimire S. Probing electron-hole coherence in strongly driven 2D materials using high-harmonic generation. Optica 2022, 9, 512 10.1364/OPTICA.444105.

[ref24] Nagai K.; Uchida K.; Kusaba S.; Endo T.; Miyata Y.; Tanaka K. Effect of incoherent electron-hole pairs on high harmonic generation in an atomically thin semiconductor. Physical Review Research 2023, 5, 043130 10.1103/PhysRevResearch.5.043130.

[ref25] Bionta M. R.; Haddad E.; Leblanc A.; Gruson V.; Lassonde P.; Ibrahim H.; Chaillou J.; Émond N.; Otto M. R.; Álvaro Jiménez-Galán; Silva R. E.; Ivanov M.; Siwick B. J.; Chaker M.; Légaré F. Tracking ultrafast solid-state dynamics using high harmonic spectroscopy. Physical Review Research 2021, 3, 023250 10.1103/PhysRevResearch.3.023250.

[ref26] Nie Z.; Guery L.; Molinero E. B.; Juergens P.; van den Hooven T. J.; Wang Y.; Jimenez Galan A.; Planken P. C. M.; Silva R. E. F.; Kraus P. M. Following the Nonthermal Phase Transition in Niobium Dioxide by Time-Resolved Harmonic Spectroscopy. Phys. Rev. Lett. 2023, 131, 243201 10.1103/PhysRevLett.131.243201. 38181131

[ref27] van der Geest M. L.; de Boer J. J.; Murzyn K.; Jürgens P.; Ehrler B.; Kraus P. M. Transient High-Harmonic Spectroscopy in an Inorganic-Organic Lead Halide Perovskite. J. Phys. Chem. Lett. 2023, 14, 10810–10818. 10.1021/acs.jpclett.3c02588. 38015825 PMC10711791

[ref28] Korobenko A.; Saha S.; Godfrey A. T.; Gertsvolf M.; Naumov A. Y.; Villeneuve D. M.; Boltasseva A.; Shalaev V. M.; Corkum P. B. High-harmonic generation in metallic titanium nitride. Nat. Commun. 2021, 12, 4981 10.1038/s41467-021-25224-z. 34404794 PMC8371016

[ref29] Heissler P.; Lugovoy E.; Hörlein R.; Waldecker L.; Wenz J.; Heigoldt M.; Khrennikov K.; Karsch S.; Krausz F.; Abel B.; et al. Using the third state of matter: high harmonic generation from liquid targets. New J. Phys. 2014, 16, 113045 10.1088/1367-2630/16/11/113045.

[ref30] Luu T. T.; Yin Z.; Jain A.; Gaumnitz T.; Pertot Y.; Ma J.; Wörner H. J. Extreme–ultraviolet high–harmonic generation in liquids. Nat. Commun. 2018, 9, 3723 10.1038/s41467-018-06040-4. 30213950 PMC6137105

[ref31] Mathijssen J.; Mazzotta Z.; Heinzerling A. M.; Eikema K. S.; Witte S. Material-specific high-order harmonic generation in laser-produced plasmas for varying plasma dynamics. Appl. Phys. B: Laser Opt. 2023, 129, 91 10.1007/s00340-023-08034-7.

[ref32] Ganeev R. High-order harmonic generation in a laser plasma: a review of recent achievements. Journal of Physics B: Atomic, Molecular and Optical Physics 2007, 40, R213 10.1088/0953-4075/40/22/R01.

[ref33] Kivshar Y. All-dielectric meta-optics and non-linear nanophotonics. National Science Review 2018, 5, 144–158. 10.1093/nsr/nwy017.

[ref34] Liu H.; Guo C.; Vampa G.; Zhang J. L.; Sarmiento T.; Xiao M.; Bucksbaum P. H.; Vučković J.; Fan S.; Reis D. A. Enhanced high-harmonic generation from an all-dielectric metasurface. Nat. Phys. 2018, 14, 1006–1010. 10.1038/s41567-018-0233-6.

[ref35] Wang L.; Kruk S.; Koshelev K.; Kravchenko I.; Luther-Davies B.; Kivshar Y. Nonlinear Wavefront Control with All-Dielectric Metasurfaces. Nano Lett. 2018, 18, 3978–3984. 10.1021/acs.nanolett.8b01460. 29749743

[ref36] Roscam Abbing S. D. C.; Kolkowski R.; Zhang Z.-Y.; Campi F.; Lötgering L.; Koenderink A. F.; Kraus P. M. Extreme-Ultraviolet Shaping and Imaging by High-Harmonic Generation from Nanostructured Silica. Phys. Rev. Lett. 2022, 128, 223902 10.1103/PhysRevLett.128.223902. 35714263

[ref37] Franz D.; Kaassamani S.; Gauthier D.; Nicolas R.; Kholodtsova M.; Douillard L.; Gomes J.-T.; Lavoute L.; Gaponov D.; Ducros N.; et al. All semiconductor enhanced high-harmonic generation from a single nanostructured cone. Sci. Rep. 2019, 9, 5663 10.1038/s41598-019-41642-y. 30952870 PMC6450872

[ref38] Zalogina A.; Carletti L.; Rudenko A.; Moloney J. V.; Tripathi A.; Lee H.-C.; Shadrivov I.; Park H.-G.; Kivshar Y.; Kruk S. S. High-harmonic generation from a subwavelength dielectric resonator. Science Advances 2023, 9, eadg2655 10.1126/sciadv.adg2655. 37126557 PMC10132744

[ref39] Dudovich N.; Smirnova O.; Levesque J.; Ivanov M.; Villeneuve D. M.; Corkum P. B. Measuring and controlling the birth of attosecond pulses. Nat. Phys. 2006, 2, 781 10.1038/nphys434.

[ref40] Roscam Abbing S.; Campi F.; Sajjadian F.; Lin N.; Smorenburg P.; Kraus P. M. Divergence Control of High-Harmonic Generation. Phys. Rev. Appl. 2020, 13, 054029 10.1103/PhysRevApplied.13.054029.

[ref41] Roscam Abbing S. D.; Campi F.; Zeltsi A.; Smorenburg P.; Kraus P. M. Divergence and efficiency optimization in polarization-controlled two-color high-harmonic generation. Sci. Rep. 2021, 11, 1–11. 10.1038/s41598-021-03657-2. 34930994 PMC8688547

[ref42] Sakai H.; Minemoto S.; Nanjo H.; Tanji H.; Suzuki T. Controlling the Orientation of Polar Molecules with Combined Electrostatic and Pulsed, Nonresonant Laser Fields. Phys. Rev. Lett. 2003, 90, 083001 10.1103/PhysRevLett.90.083001. 12633422

[ref43] Rupenyan A.; Kraus P. M.; Schneider J.; Wörner H. J. High-harmonic spectroscopy of isoelectronic molecules: Wavelength scaling of electronic-structure and multielectron effects. Phys. Rev. A 2013, 87, 033409 10.1103/PhysRevA.87.033409.

[ref44] Frumker E.; Hebeisen C. T.; Kajumba N.; Bertrand J. B.; Wörner H. J.; Spanner M.; Villeneuve D. M.; Naumov A.; Corkum P. B. Oriented Rotational Wave-Packet Dynamics Studies via High Harmonic Generation. Phys. Rev. Lett. 2012, 109, 113901 10.1103/PhysRevLett.109.113901. 23005628

[ref45] Kraus P. M.; Rupenyan A.; Wörner H. J. High-harmonic spectroscopy of oriented OCS molecules: emission of even and odd harmonics. Phys. Rev. Lett. 2012, 109, 233903 10.1103/PhysRevLett.109.233903. 23368204

[ref46] Kraus P. M.; Baykusheva D.; Wörner H. J. Two-pulse orientation dynamics and high-harmonic spectroscopy of strongly oriented molecules. J. Phys. B 2014, 47, 124030 10.1088/0953-4075/47/12/124030.

[ref47] Kraus P. M.; Tolstikhin O. I.; Baykusheva D.; Rupenyan A.; Schneider J.; Bisgaard C. Z.; Morishita T.; Jensen F.; Madsen L. B.; Wörner H. J. Observation of laser-induced electronic structure in oriented polyatomic molecules. Nat. Commun. 2015, 6, 7039 10.1038/ncomms8039. 25940229 PMC4432593

[ref48] Wörner H. J.; Bertrand J. B.; Kartashov D. V.; Corkum P. B.; Villeneuve D. M. Following a chemical reaction using high-harmonic interferometry. Nature 2010, 466, 604–607. 10.1038/nature09185. 20671706

[ref49] Kraus P. M.; Arasaki Y.; Bertrand J. B.; Patchkovskii S.; Corkum P. B.; Villeneuve D. M.; Takatsuka K.; Wörner H. J. Time-resolved high-harmonic spectroscopy of nonadiabatic dynamics in NO_2_. Phys. Rev. A 2012, 85, 043409 10.1103/PhysRevA.85.043409.

[ref50] Ruf H.; et al. High-harmonic transient grating spectroscopy of NO_2_ electronic relaxation. J. Chem. Phys. 2012, 137, 224303 10.1063/1.4768810. 23248999

[ref51] Kraus P. M.; Wörner H. J. Time-resolved high-harmonic spectroscopy of valence electron dynamics. Chem. Phys. 2013, 414, 32–44. 10.1016/j.chemphys.2012.01.013.

[ref52] Kraus P. M.; Zhang S. B.; Gijsbertsen A.; Lucchese R. R.; Rohringer N.; Wörner H. J. High-Harmonic Probing of Electronic Coherence in Dynamically Aligned Molecules. Phys. Rev. Lett. 2013, 111, 243005 10.1103/PhysRevLett.111.243005. 24483654

[ref53] Baykusheva D.; Kraus P. M.; Zhang S. B.; Rohringer N.; Wörner H. J. The sensitivities of high-harmonic generation and strong-field ionization to coupled electronic and nuclear dynamics. Faraday Discuss. 2014, 171, 113 10.1039/C4FD00018H. 25415558

[ref54] Zhang S. B.; Baykusheva D.; Kraus P. M.; Wörner H. J.; Rohringer N. Theoretical study of molecular electronic and rotational coherences by high-order-harmonic generation. Phys. Rev. A 2015, 91, 023421 10.1103/PhysRevA.91.023421.

[ref55] Hohenleutner M.; Langer F.; Schubert O.; Knorr M.; Huttner U.; Koch S. W.; Kira M.; Huber R. Real-time observation of interfering crystal electrons in high-harmonic generation. Nature 2015, 523, 572–575. 10.1038/nature14652. 26223624

[ref56] Abbing S. D. R.; Kolkowski R.; Zhang Z.-Y.; Campi F.; Lötgering L.; Koenderink A. F.; Kraus P. M. Extreme-ultraviolet shaping and imaging by high-harmonic generation from nanostructured silica. Physical Review Letters 2022, 128, 223902 10.1103/PhysRevLett.128.223902. 35714263

[ref57] Korobenko A.; Rashid S.; Heide C.; Naumov A. Y.; Reis D. A.; Berini P.; Corkum P. B.; Vampa G. In-Situ Nanoscale Focusing of Extreme Ultraviolet Solid-State High Harmonics. Physical Review X 2022, 12, 041036 10.1103/PhysRevX.12.041036.

[ref58] Murzyn K.; van der Geest M. L. S.; Guery L.; Nie Z.; van Essen P.; Witte S.; Kraus P. M. Breaking Abbe’s diffraction limit with harmonic deactivation microscopy.. arXiv:2403.06617 [physics.optics] 2024, na 10.48550/arXiv.2403.06617. PMC11559608 39536111

[ref59] Nishidome H.; Nagai K.; Uchida K.; Ichinose Y.; Yomogida Y.; Miyata Y.; Tanaka K.; Yanagi K. Control of High-Harmonic Generation by Tuning the Electronic Structure and Carrier Injection. Nano Lett. 2020, 20, 6215–6221. 10.1021/acs.nanolett.0c02717. 32787188

[ref60] Udono M.; Sugimoto K.; Kaneko T.; Ohta Y. Excitonic effects on high-harmonic generation in Mott insulators. Phys. Rev. B 2022, 105, L241108 10.1103/PhysRevB.105.L241108.

[ref61] Molinero E. B.; Amorim B.; Malakhov M.; Cistaro G.; Álvaro Jiménez-Galán; Ivanov M.; Picón A.; San-José P.; Silva R. E. F. Formation, stability, and highly nonlinear optical response of excitons to intense light fields interacting with two-dimensional materials. arXiv:2307.16647 2023, na 10.48550/arXiv.2307.16647.

[ref62] Wen X.; Xu W.; Zhao W.; Khurgin J. B.; Xiong Q. Plasmonic Hot Carriers-Controlled Second Harmonic Generation in WSe2 Bilayers. Nano Lett. 2018, 18, 1686–1692. 10.1021/acs.nanolett.7b04707. 29376381

[ref63] Soavi G.; Wang G.; Rostami H.; Tomadin A.; Balci O.; Paradisanos I.; Pogna E. A.; Cerullo G.; Lidorikis E.; Polini M.; Ferrari A. C. Hot Electrons Modulation of Third-Harmonic Generation in Graphene. ACS Photonics 2019, 6, 2841–2849. 10.1021/acsphotonics.9b00928.

[ref64] Cox J. D.; Marini A.; Abajo F. J. G. D. Plasmon-assisted high-harmonic generation in graphene. Nat. Commun. 2017, 8, 14380 10.1038/ncomms14380. 28224998 PMC5322527

[ref65] Pattanayak A.; Mrudul M. S.; Dixit G. Influence of vacancy defects in solid high-order harmonic generation. Phys. Rev. A 2020, 101, 013404 10.1103/PhysRevA.101.013404.

[ref66] Xu S.; Yu J.; Ye C.; Zhang H.; Wang Z.; Hu J. The defect-state-assisted enhancement of high harmonic generation in bulk ZnO. Appl. Phys. Lett. 2023, 122, 182105 10.1063/5.0145728.

[ref67] Nefedova V. E.; et al. Enhanced extreme ultraviolet high-harmonic generation from chromium-doped magnesium oxide. Appl. Phys. Lett. 2021, 118, 201103 10.1063/5.0047421.

[ref68] Jia L.; Zhang Z.; Yang D. Z.; Liu Y.; Si M. S.; Zhang G. P.; Liu Y. S. Optical high-order harmonic generation as a structural characterization tool. Phys. Rev. B 2020, 101, 144304 10.1103/PhysRevB.101.144304.

[ref69] Liu H.; Li Y.; You Y. S.; Ghimire S.; Heinz T. F.; Reis D. A. High-harmonic generation from an atomically thin semiconductor. Nat. Phys. 2017, 13, 262–265. 10.1038/nphys3946.

[ref70] Luu T. T.; Wörner H. J. Measurement of the Berry curvature of solids using high-harmonic spectroscopy. Nat. Commun. 2018, 9, 916 10.1038/s41467-018-03397-4. 29500349 PMC5834542

[ref71] Luu T. T.; Wörner H. J. Observing broken inversion symmetry in solids using two-color high-order harmonic spectroscopy. Phys. Rev. A 2018, 98, 041802 10.1103/PhysRevA.98.041802.

[ref72] Ward J. F. Calculation of Nonlinear Optical Susceptibilities Using Diagrammatic Perturbation Theory I. Introduction. Rev. Mod. Phys. 1965, 37, 1 10.1103/RevModPhys.37.1.

[ref73] Yue L.; Gaarde M. B. Introduction to theory of high-harmonic generation in solids: tutorial. Journal of the Optical Society of America B 2022, 39, 535–555. 10.1364/JOSAB.448602.

[ref74] Vampa G.; McDonald C. R.; Orlando G.; Klug D. D.; Corkum P. B.; Brabec T. Theoretical analysis of high-harmonic generation in solids. Phys. Rev. Lett. 2014, 113, 073901 10.1103/PhysRevLett.113.073901. 25170708

[ref75] Lindberg M.; Koch S. W. Effective Bloch equations for semiconductors. Phys. Rev. B 1988, 38, 3342 10.1103/PhysRevB.38.3342. 9946675

[ref76] Haug H.; Koch S. W. Quantum Theory of the Optical and Electronic Properties of Semiconductors; World Scientific, 2004; p 453, 10.1142/7184.

[ref77] Brown G. G.; Álvaro Jiménez-Galán; Silva R. E. F.; Ivanov M. A Real-Space Perspective on Dephasing in Solid-State High Harmonic Generation. arXiv:2210.16889 2022, na 10.48550/arXiv.2210.16889.

[ref78] Mrudul M. S.; Pattanayak A.; Ivanov M.; Dixit G. Direct numerical observation of real-space recollision in high-order harmonic generation from solids. Phys. Rev. A 2019, 100, 043420 10.1103/PhysRevA.100.043420.

[ref79] Margalit Y.; Lu Y.-K.; Top F. C.; Ketterle W. Pauli blocking of light scattering in degenerate fermions. Science 2021, 374, 976–979. 10.1126/science.abi6153. 34793214

[ref80] Chong S.; Min W.; Xie X. S. Ground-state depletion microscopy: Detection sensitivity of single-molecule optical absorption at room temperature. J. Phys. Chem. Lett. 2010, 1, 3316–3322. 10.1021/jz1014289.

[ref81] Jannin R.; van der Werf Y.; Steinebach K.; Bethlem H. L.; Eikema K. S. Pauli blocking of stimulated emission in a degenerate Fermi gas. Nat. Commun. 2022, 13, 6479 10.1038/s41467-022-34135-6. 36309519 PMC9617905

[ref82] Kastner M. A. The single-electron transistor. Rev. Mod. Phys. 1992, 64, 849 10.1103/RevModPhys.64.849.

[ref83] Becker P. C.; Fragnito H. L.; Cruz C. H. B.; Fork R. L.; Cunningham J. E.; Henry J. E.; Shank C. U. Femtosecond Photon Echoes from Band-to-Band Transitions in GaAs. Phys. Rev. Lett. 1988, 61, 1647 10.1103/PhysRevLett.61.1647. 10038859

[ref84] Kilen I.; Kolesik M.; Hader J.; Moloney J. V.; Huttner U.; Hagen M. K.; Koch S. W. Propagation Induced Dephasing in Semiconductor High-Harmonic Generation. Phys. Rev. Lett. 2020, 125, 083901 10.1103/PhysRevLett.125.083901. 32909805

[ref85] Vampa G.; McDonald C.; Orlando G.; Klug D.; Corkum P.; Brabec T. Theoretical analysis of high-harmonic generation in solids. Physical review letters 2014, 113, 073901 10.1103/PhysRevLett.113.073901. 25170708

[ref86] Tancogne-Dejean N.; Mücke O. D.; Kärtner F. X.; Rubio A. Ellipticity dependence of high-harmonic generation in solids originating from coupled intraband and interband dynamics. Nat. Commun. 2017, 8, 745 10.1038/s41467-017-00764-5. 28963478 PMC5622149

[ref87] Bennett B. R.; Soref R. A.; Del Alamo J. A. Carrier-induced change in refractive index of InP, GaAs and InGaAsP. IEEE J. Quantum Electron. 1990, 26, 113–122. 10.1109/3.44924.

[ref88] Imada M.; Fujimori A.; Tokura Y. Metal-insulator transitions. Reviews of modern physics 1998, 70, 1039 10.1103/RevModPhys.70.1039.

[ref89] Wegkamp D.; Stähler J. Ultrafast dynamics during the photoinduced phase transition in VO2. Prog. Surf. Sci. 2015, 90, 464–502. 10.1016/j.progsurf.2015.10.001.

[ref90] Wegkamp D.; Herzog M.; Xian L.; Gatti M.; Cudazzo P.; McGahan C. L.; Marvel R. E.; Haglund Jr R. F.; Rubio A.; Wolf M.; et al. Instantaneous band gap collapse in photoexcited monoclinic VO 2 due to photocarrier doping. Physical review letters 2014, 113, 216401 10.1103/PhysRevLett.113.216401. 25479507

[ref91] Wang Y.; Nie Z.; Shi Y.; Wang Y.; Wang F. Coherent vibrational dynamics of NbO 2 film. Physical Review Materials 2022, 6, 035005 10.1103/PhysRevMaterials.6.035005.

[ref92] Yang Z.; Ko C.; Ramanathan S. Oxide electronics utilizing ultrafast metal-insulator transitions. Annu. Rev. Mater. Res. 2011, 41, 337–367. 10.1146/annurev-matsci-062910-100347.

[ref93] Kumar S.; Williams R. S.; Wang Z. Third-order nanocircuit elements for neuromorphic engineering. Nature 2020, 585, 518–523. 10.1038/s41586-020-2735-5. 32968256

[ref94] Li X.; Fan J.; Ma J.; Wang G.; Jin C. Application of optimized waveforms for enhancing high-harmonic yields in a three-color laser-field synthesizer. Opt. Express 2019, 27, 841 10.1364/OE.27.000841. 30696164

[ref95] Kroh T.; Jin C.; Krogen P.; Keathley P. D.; Calendron A.-L.; Siqueira J. P.; Liang H.; Falcão-Filho E. L.; Lin C. D.; Kärtner F. X.; Hong K.-H. Enhanced high-harmonic generation up to the soft X-ray region driven by mid-infrared pulses mixed with their third harmonic. Opt. Express 2018, 26, 16955 10.1364/OE.26.016955. 30119513

[ref96] Liu C.; Zheng Y.; Zeng Z.; Li R. Effect of elliptical polarization of driving field on high-order-harmonic generation in semiconductor ZnO. Phys. Rev. A 2016, 93, 043806 10.1103/PhysRevA.93.043806.

[ref97] Hollinger R.; Herrmann P.; Korolev V.; Zapf M.; Shumakova V.; Röder R.; Uschmann I.; Pugžlys A.; Baltuška A.; Zürch M.; Ronning C.; Spielmann C.; Kartashov D. Polarization dependent excitation and high harmonic generation from intense mid-IR laser pulses in ZnO. Nanomaterials 2021, 11, 4 10.3390/nano11010004. PMC7822178 33375116

[ref98] Rodnyi P. A.; Khodyuk I. V. Optical and Luminescence Properties of Zinc Oxide. Optics and spectroscopy 2011, 111, 776–785. 10.1134/S0030400X11120216.

